# 4-Chloro-*N*-(2-chloro­phen­yl)-2-methyl­benzene­sulfonamide

**DOI:** 10.1107/S1600536809007880

**Published:** 2009-03-11

**Authors:** B. Thimme Gowda, Sabine Foro, P. G. Nirmala, K. S. Babitha, Hartmut Fuess

**Affiliations:** a Department of Chemistry, Mangalore University, Mangalagangotri 574 199, Mangalore, India; b Institute of Materials Science, Darmstadt University of Technology, Petersenstrasse 23, D-64287 Darmstadt, Germany

## Abstract

In the crystal structure of the title compound, C_13_H_11_Cl_2_NO_2_S, the conformations of the N—C bond in the C—SO_2_—NH—C segment are *trans* and *gauche* with respect to the S=O bonds. The C—S(O_2_)—N(H)—C torsion angle is 74.8 (4)°, indicating that the mol­ecule is bent at the S atom. In the crystal structure, inversion dimers linked by pairs of N—H⋯O hydrogen bonds occur. An intramolecular N—H⋯Cl inter­action is also present.

## Related literature

For related structures of *N*-(ar­yl)-aryl­sulfonamides, see: Gelbrich *et al.* (2007 ▶); Gowda *et al.* (2009**a* ▶,b* ▶); Perlovich *et al.* (2006 ▶).
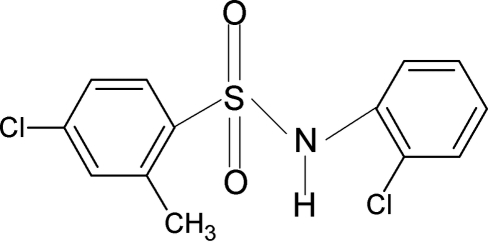


## Experimental

### 

#### Crystal data

C_13_H_11_Cl_2_NO_2_S
*M* *_r_* = 316.19
Triclinic, 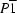

*a* = 8.089 (2) Å
*b* = 8.096 (2) Å
*c* = 10.946 (3) Å
α = 96.00 (1)°
β = 97.11 (2)°
γ = 105.67 (2)°
*V* = 677.7 (3) Å^3^
*Z* = 2
Cu *K*α radiation
μ = 5.73 mm^−1^
*T* = 299 K
0.45 × 0.33 × 0.08 mm

#### Data collection

Enraf–Nonius CAD-4 diffractometer
Absorption correction: ψ scan (North *et al.*, 1968 ▶) *T*_min_ = 0.150, *T*_max_ = 0.640
2684 measured reflections
2414 independent reflections
1932 reflections with *I* > 2σ(*I*)
*R*_int_ = 0.037
3 standard reflections frequency: 120 min intensity decay: 1.0%

#### Refinement

*R*[*F* ^2^ > 2σ(*F* ^2^)] = 0.083
*wR*(*F* ^2^) = 0.236
*S* = 1.05
2414 reflections
177 parameters
H atoms treated by a mixture of independent and constrained refinement
Δρ_max_ = 0.60 e Å^−3^
Δρ_min_ = −0.67 e Å^−3^

### 

Data collection: *CAD-4-PC* (Enraf–Nonius, 1996 ▶); cell refinement: *CAD-4-PC*; data reduction: *REDU4* (Stoe & Cie, 1987 ▶); program(s) used to solve structure: *SHELXS97* (Sheldrick, 2008 ▶); program(s) used to refine structure: *SHELXL97* (Sheldrick, 2008 ▶); molecular graphics: *PLATON* (Spek, 2009 ▶); software used to prepare material for publication: *SHELXL97*.

## Supplementary Material

Crystal structure: contains datablocks I, global. DOI: 10.1107/S1600536809007880/tk2384sup1.cif

Structure factors: contains datablocks I. DOI: 10.1107/S1600536809007880/tk2384Isup2.hkl

Additional supplementary materials:  crystallographic information; 3D view; checkCIF report

## Figures and Tables

**Table 1:** Hydrogen-bond geometry (Å, °)

*D*—H⋯*A*	*D*—H	H⋯*A*	*D*⋯*A*	*D*—H⋯*A*
N1—H1*N*⋯O2^i^	0.88 (5)	2.17 (5)	2.994 (5)	157 (4)
N1—H1*N*⋯Cl2	0.88 (5)	2.67 (5)	3.011 (4)	104 (4)

Symmetry code: (i) 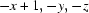.

**Fig. 1.**
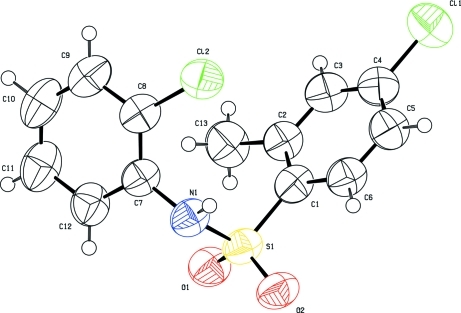
Molecular structure of (I), showing the atom labeling scheme. The displacement ellipsoids are drawn at the 50% probability level. The H atoms are represented as small spheres of arbitrary radii.

**Fig. 2.**
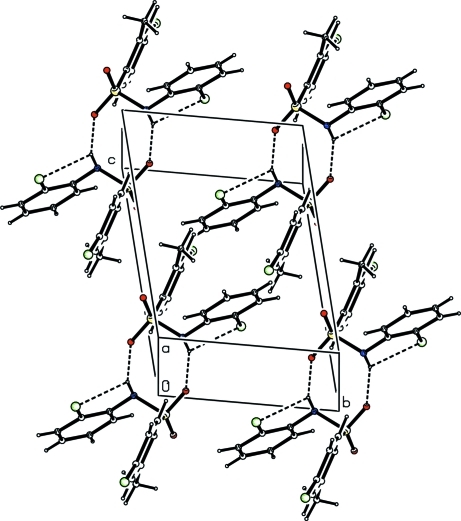
Molecular packing of (I) with hydrogen bonding shown as dashed lines.

## supplementary crystallographic information

### Comment

In the present work, as part of a study of substituent effects on the structures of *N*-(aryl)-arylsulfonamides (Gowda *et al.* 2008*a*,*b*, 2009), the structure of 2-methyl-4-chloro-*N*-(2-chlorophenyl)benzenesulfonamide (I) has been determined. The conformations of the N—C bond in the C—S(O_2_)—N(H)—C segment are *trans* and *gauche* with respect to the S=O bonds (Fig. 1). The torsion angle of C1—S1—N1—C1 is 74.8 (4)°, indicating the molecule is bent at the S1 atom. The two benzene rings are tilted relative to each other by 45.5 (2)°, compared with the values of 86.6 (2)° (molecule 1) and 83.0 (2)° (molecule 2), in the two independent molecules of 2-methyl-4-chloro-*N*-(phenyl)-benzenesulfonamide (II) (Gowda *et al.*, 2009). Bond distance parameters in (I) are similar to those observed in (II), 2,4-dimethyl-*N*-(phenyl)benzenesulfonamide (Gowda *et al.*, 2008*b*) and other aryl sulfonamides (Perlovich *et al.*, 2006; Gelbrich *et al.*, 2007). The crystal structure comprises the packing of centrosymmetric molecules connected via N—H···O hydrogen bonds (Table 1 & Fig. 2).

### Experimental

*m*-Chlorotoluene (10 ml) in chloroform (40 ml) was treated dropwise with chlorosulfonic acid (25 ml) at 0° C. After the initial evolution of HCl subsided, the reaction mixture was brought to room temperature and poured into crushed ice in a beaker. The chloroform layer was separated, washed with cold water and allowed to evaporate slowly. The residual 2-methyl-4-chlorobenzenesulfonylchloride was treated with 2-chloroaniline in a stoichiometric ratio and boiled for ten minutes. The reaction mixture was then cooled to room temperature and added to ice cold water (100 ml). The resultant solid 2-methyl-4-chloro-*N*-(2-chlorophenyl)-benzenesulfonamide was filtered under suction and washed thoroughly with cold water. It was then recrystallized to constant melting point from dilute ethanol. The purity of the compound was checked and characterized by recording its infrared and NMR spectra. The single crystals used in X-ray diffraction studies were grown from an ethanolic solution by slow evaporation at room temperature.

### Refinement

The N-bound H atom was located in difference map and its positional parameters refined; N—H = 0.88 (5) Å. The remaining H atoms were positioned with idealized geometries using a riding model with C—H = 0.93–0.96 Å. All H atoms were refined with isotropic displacement parameters set to 1.2 x *U*_eq_(parent atom).

### Crystal data

**Table:**

C_13_H_11_Cl_2_NO_2_S	*Z* = 2
*M**_r_* = 316.19	*F*(000) = 324
Triclinic, *P*1	*D*_x_ = 1.549 Mg m^−^^3^
Hall symbol: -P 1	Cu *K*α radiation, λ = 1.54180 Å
*a* = 8.089 (2) Å	Cell parameters from 25 reflections
*b* = 8.096 (2) Å	θ = 5.7–19.7°
*c* = 10.946 (3) Å	µ = 5.73 mm^−^^1^
α = 96.00 (1)°	*T* = 299 K
β = 97.11 (2)°	Plate, colourless
γ = 105.67 (2)°	0.45 × 0.33 × 0.08 mm
*V* = 677.7 (3) Å^3^	

### Data collection

**Table:**

Enraf–Nonius CAD-4 diffractometer	1932 reflections with *I* > 2σ(*I*)
Radiation source: fine-focus sealed tube	*R*_int_ = 0.037
graphite	θ_max_ = 66.9°, θ_min_ = 4.1°
ω scans	*h* = −9→1
Absorption correction: ψ scan (North *et al.*, 1968)	*k* = −9→9
*T*_min_ = 0.150, *T*_max_ = 0.640	*l* = −12→13
2684 measured reflections	3 standard reflections every 120 min
2414 independent reflections	intensity decay: 1.0%

### Refinement

**Table:**

Refinement on *F*^2^	Secondary atom site location: difference Fourier map
Least-squares matrix: full	Hydrogen site location: inferred from neighbouring sites
*R*[*F*^2^ > 2σ(*F*^2^)] = 0.083	H atoms treated by a mixture of independent and constrained refinement
*wR*(*F*^2^) = 0.236	*w* = 1/[σ^2^(*F*_o_^2^) + (0.179*P*)^2^ + 0.1079*P*] where *P* = (*F*_o_^2^ + 2*F*_c_^2^)/3
*S* = 1.05	(Δ/σ)_max_ = 0.008
2414 reflections	Δρ_max_ = 0.60 e Å^−^^3^
177 parameters	Δρ_min_ = −0.67 e Å^−^^3^
0 restraints	Extinction correction: *SHELXL97* (Sheldrick, 2008), Fc^*^=kFc[1+0.001xFc^2^λ^3^/sin(2θ)]^-1/4^
Primary atom site location: structure-invariant direct methods	Extinction coefficient: 0.039 (6)

### Special details

**Table:**

Geometry. All e.s.d.'s (except the e.s.d. in the dihedral angle between two l.s. planes) are estimated using the full covariance matrix. The cell e.s.d.'s are taken into account individually in the estimation of e.s.d.'s in distances, angles and torsion angles; correlations between e.s.d.'s in cell parameters are only used when they are defined by crystal symmetry. An approximate (isotropic) treatment of cell e.s.d.'s is used for estimating e.s.d.'s involving l.s. planes.
Refinement. Refinement of *F*^2^ against ALL reflections. The weighted *R*-factor *wR* and goodness of fit *S* are based on *F*^2^, conventional *R*-factors *R* are based on *F*, with *F* set to zero for negative *F*^2^. The threshold expression of *F*^2^ > σ(*F*^2^) is used only for calculating *R*-factors(gt) *etc*. and is not relevant to the choice of reflections for refinement. *R*-factors based on *F*^2^ are statistically about twice as large as those based on *F*, and *R*- factors based on ALL data will be even larger.

### Fractional atomic coordinates and isotropic or equivalent isotropic displacement parameters (Å^2^)

**Table:**

	*x*	*y*	*z*	*U*_iso_*/*U*_eq_	
Cl1	0.35954 (18)	0.30027 (19)	0.56480 (12)	0.0932 (5)	
Cl2	0.70514 (17)	0.48939 (15)	0.15780 (13)	0.0901 (5)	
S1	0.71199 (12)	−0.00409 (12)	0.15693 (9)	0.0651 (4)	
O1	0.8667 (4)	−0.0424 (4)	0.2054 (3)	0.0761 (8)	
O2	0.5732 (4)	−0.1359 (4)	0.0795 (3)	0.0757 (8)	
N1	0.7673 (4)	0.1494 (4)	0.0701 (3)	0.0659 (9)	
H1N	0.678 (7)	0.178 (6)	0.034 (5)	0.079*	
C1	0.6225 (5)	0.0807 (5)	0.2792 (4)	0.0637 (9)	
C2	0.7215 (5)	0.1757 (5)	0.3907 (4)	0.0660 (10)	
C3	0.6344 (6)	0.2423 (6)	0.4761 (4)	0.0719 (11)	
H3	0.6974	0.3069	0.5507	0.086*	
C4	0.4585 (6)	0.2158 (6)	0.4536 (4)	0.0708 (10)	
C5	0.3607 (6)	0.1217 (6)	0.3426 (4)	0.0732 (11)	
H5	0.2410	0.1036	0.3271	0.088*	
C6	0.4441 (5)	0.0565 (5)	0.2569 (4)	0.0686 (10)	
H6	0.3800	−0.0057	0.1818	0.082*	
C7	0.9177 (5)	0.2931 (5)	0.1116 (4)	0.0625 (9)	
C8	0.9064 (6)	0.4560 (5)	0.1521 (4)	0.0693 (10)	
C9	1.0553 (7)	0.5950 (6)	0.1862 (4)	0.0812 (12)	
H9	1.0466	0.7053	0.2114	0.097*	
C10	1.2140 (6)	0.5692 (7)	0.1826 (5)	0.0900 (15)	
H10	1.3139	0.6619	0.2069	0.108*	
C11	1.2280 (6)	0.4087 (7)	0.1436 (5)	0.0900 (15)	
H11	1.3372	0.3921	0.1427	0.108*	
C12	1.0796 (6)	0.2698 (6)	0.1052 (5)	0.0760 (11)	
H12	1.0891	0.1615	0.0753	0.091*	
C13	0.9170 (6)	0.2092 (8)	0.4254 (4)	0.0875 (14)	
H13A	0.9430	0.1011	0.4307	0.105*	
H13B	0.9567	0.2823	0.5044	0.105*	
H13C	0.9747	0.2655	0.3630	0.105*	

### Atomic displacement parameters (Å^2^)

**Table:**

	*U*^11^	*U*^22^	*U*^33^	*U*^12^	*U*^13^	*U*^23^
Cl1	0.0896 (9)	0.1076 (10)	0.0887 (9)	0.0410 (7)	0.0150 (6)	0.0072 (7)
Cl2	0.0869 (8)	0.0730 (8)	0.1186 (10)	0.0325 (6)	0.0255 (7)	0.0141 (6)
S1	0.0608 (6)	0.0547 (6)	0.0758 (7)	0.0165 (4)	−0.0011 (4)	0.0066 (4)
O1	0.0695 (18)	0.0690 (17)	0.093 (2)	0.0285 (14)	0.0023 (15)	0.0136 (14)
O2	0.0690 (17)	0.0601 (16)	0.0872 (19)	0.0119 (13)	−0.0024 (14)	−0.0020 (13)
N1	0.0591 (18)	0.0635 (19)	0.0692 (19)	0.0144 (14)	−0.0032 (14)	0.0074 (14)
C1	0.058 (2)	0.060 (2)	0.073 (2)	0.0185 (16)	−0.0008 (17)	0.0152 (17)
C2	0.061 (2)	0.071 (2)	0.065 (2)	0.0205 (17)	−0.0021 (17)	0.0108 (17)
C3	0.072 (2)	0.073 (2)	0.065 (2)	0.0210 (19)	−0.0043 (18)	0.0072 (18)
C4	0.075 (2)	0.070 (2)	0.071 (2)	0.0278 (19)	0.0071 (19)	0.0153 (18)
C5	0.062 (2)	0.079 (3)	0.079 (3)	0.0260 (19)	0.0022 (19)	0.013 (2)
C6	0.061 (2)	0.067 (2)	0.072 (2)	0.0168 (18)	−0.0026 (18)	0.0084 (17)
C7	0.058 (2)	0.062 (2)	0.063 (2)	0.0149 (16)	−0.0013 (15)	0.0093 (15)
C8	0.072 (2)	0.065 (2)	0.068 (2)	0.0178 (18)	0.0016 (18)	0.0123 (17)
C9	0.087 (3)	0.067 (2)	0.077 (3)	0.006 (2)	0.000 (2)	0.0105 (19)
C10	0.070 (3)	0.086 (3)	0.095 (3)	−0.005 (2)	−0.011 (2)	0.025 (2)
C11	0.061 (2)	0.094 (3)	0.109 (4)	0.014 (2)	−0.005 (2)	0.032 (3)
C12	0.062 (2)	0.080 (3)	0.089 (3)	0.023 (2)	0.008 (2)	0.022 (2)
C13	0.062 (3)	0.114 (4)	0.075 (3)	0.022 (2)	−0.008 (2)	−0.003 (2)

### Geometric parameters (Å, °)

**Table:**

Cl1—C4	1.719 (5)	C5—H5	0.9300
Cl2—C8	1.728 (5)	C6—H6	0.9300
S1—O1	1.423 (3)	C7—C8	1.378 (6)
S1—O2	1.430 (3)	C7—C12	1.381 (6)
S1—N1	1.643 (4)	C8—C9	1.388 (6)
S1—C1	1.761 (4)	C9—C10	1.360 (8)
N1—C7	1.424 (5)	C9—H9	0.9300
N1—H1N	0.88 (5)	C10—C11	1.364 (8)
C1—C6	1.390 (5)	C10—H10	0.9300
C1—C2	1.398 (5)	C11—C12	1.389 (6)
C2—C3	1.387 (7)	C11—H11	0.9300
C2—C13	1.522 (6)	C12—H12	0.9300
C3—C4	1.366 (6)	C13—H13A	0.9600
C3—H3	0.9300	C13—H13B	0.9600
C4—C5	1.388 (6)	C13—H13C	0.9600
C5—C6	1.364 (7)		
			
O1—S1—O2	120.08 (19)	C1—C6—H6	119.2
O1—S1—N1	107.27 (18)	C8—C7—C12	119.1 (4)
O2—S1—N1	104.75 (18)	C8—C7—N1	122.1 (4)
O1—S1—C1	109.99 (19)	C12—C7—N1	118.8 (4)
O2—S1—C1	106.96 (19)	C7—C8—C9	120.6 (4)
N1—S1—C1	107.03 (18)	C7—C8—Cl2	120.0 (3)
C7—N1—S1	120.4 (3)	C9—C8—Cl2	119.4 (4)
C7—N1—H1N	114 (3)	C10—C9—C8	119.6 (5)
S1—N1—H1N	113 (3)	C10—C9—H9	120.2
C6—C1—C2	120.1 (4)	C8—C9—H9	120.2
C6—C1—S1	116.1 (3)	C9—C10—C11	120.6 (4)
C2—C1—S1	123.7 (3)	C9—C10—H10	119.7
C3—C2—C1	117.4 (4)	C11—C10—H10	119.7
C3—C2—C13	118.0 (4)	C10—C11—C12	120.2 (5)
C1—C2—C13	124.6 (4)	C10—C11—H11	119.9
C4—C3—C2	121.9 (4)	C12—C11—H11	119.9
C4—C3—H3	119.0	C7—C12—C11	119.8 (5)
C2—C3—H3	119.0	C7—C12—H12	120.1
C3—C4—C5	120.6 (4)	C11—C12—H12	120.1
C3—C4—Cl1	119.2 (4)	C2—C13—H13A	109.5
C5—C4—Cl1	120.3 (4)	C2—C13—H13B	109.5
C6—C5—C4	118.5 (4)	H13A—C13—H13B	109.5
C6—C5—H5	120.8	C2—C13—H13C	109.5
C4—C5—H5	120.8	H13A—C13—H13C	109.5
C5—C6—C1	121.6 (4)	H13B—C13—H13C	109.5
C5—C6—H6	119.2		
			
O1—S1—N1—C7	−43.2 (4)	Cl1—C4—C5—C6	−179.9 (3)
O2—S1—N1—C7	−171.9 (3)	C4—C5—C6—C1	0.7 (6)
C1—S1—N1—C7	74.8 (4)	C2—C1—C6—C5	−0.9 (6)
O1—S1—C1—C6	−153.8 (3)	S1—C1—C6—C5	−177.6 (3)
O2—S1—C1—C6	−21.8 (3)	S1—N1—C7—C8	−106.8 (4)
N1—S1—C1—C6	90.0 (3)	S1—N1—C7—C12	76.4 (5)
O1—S1—C1—C2	29.7 (4)	C12—C7—C8—C9	−0.1 (6)
O2—S1—C1—C2	161.6 (3)	N1—C7—C8—C9	−176.9 (4)
N1—S1—C1—C2	−86.6 (4)	C12—C7—C8—Cl2	178.6 (3)
C6—C1—C2—C3	0.2 (6)	N1—C7—C8—Cl2	1.9 (6)
S1—C1—C2—C3	176.7 (3)	C7—C8—C9—C10	−1.7 (7)
C6—C1—C2—C13	179.5 (4)	Cl2—C8—C9—C10	179.6 (4)
S1—C1—C2—C13	−4.0 (6)	C8—C9—C10—C11	1.2 (8)
C1—C2—C3—C4	0.6 (6)	C9—C10—C11—C12	1.0 (8)
C13—C2—C3—C4	−178.8 (4)	C8—C7—C12—C11	2.3 (6)
C2—C3—C4—C5	−0.7 (7)	N1—C7—C12—C11	179.2 (4)
C2—C3—C4—Cl1	179.2 (3)	C10—C11—C12—C7	−2.7 (7)
C3—C4—C5—C6	0.1 (6)		

### Hydrogen-bond geometry (Å, °)

**Table:**

*D*—H···*A*	*D*—H	H···*A*	*D*···*A*	*D*—H···*A*
N1—H1N···O2^i^	0.88 (5)	2.17 (5)	2.994 (5)	157 (4)
N1—H1N···Cl2	0.88 (5)	2.67 (5)	3.011 (4)	104 (4)

Symmetry codes: (i) −*x*+1, −*y*, −*z*.
